# Effect of Gender on Meat Quality from Adult Obsolescent Horses

**DOI:** 10.3390/ani11102880

**Published:** 2021-10-01

**Authors:** Violeta Razmaitė, Rūta Šveistienė, Alma Račkauskaitė, Virginija Jatkauskienė

**Affiliations:** Animal Science Institute, Lithuanian University of Health Sciences, R. Žebenkos 12, LT-82317 Baisogala, Lithuania; Ruta.Sveistiene@lsmuni.lt (R.Š.); Alma.Rackauskaite@lsmuni.lt (A.R.); Virginija.Jatkauskiene@lsmuni.lt (V.J.)

**Keywords:** horsemeat, quality properties, fatty acids, lipid indices

## Abstract

**Simple Summary:**

Horses have played an important role for humans through history, being used as a transport, draught power in the past, and they continue to be used for leisure activities, recreation, and other tasks, including horse milk and meat consumption. Different contradictory cultural meanings are associated with horse meat consumption, and horse meat is a taboo in some countries but remains popular in others. There are also efforts to promote horse meat consumption as a healthy and nutritious food. As only a small part of raised horses is destined for meat production, in Europe most of horses are usually not slaughtered but are put down in other ways and often destroyed. Therefore, the objective of this study was to assess the influence of gender, age, and carcass weight on the properties of meat from adult obsolescent horses. Age did not show any effect on meat properties, whereas gender appeared to affect intramuscular fat, cholesterol content, color parameters, fatty acid composition, and toughness. Despite the differences in meat quality parameters between the genders, horse meat from all groups demonstrated a good quality, favorable fatty acid composition, and lipid indices in relation to healthy nutrition.

**Abstract:**

The objective of the study was to assess the influence of gender, age, and carcass weight on the properties of meat from adult horses slaughtered in Lithuania. *M. pectoralis profundus* of twenty-six obsolescent horses from 3 to 21 years of age were used in the experiment. Gender appeared to affect the horse meat properties. Stallions demonstrated (*p* < 0.01 and *p* < 0.05, respectively) lower content of dry matter and also considerably lower (*p* < 0.001 and *p* < 0.01) intramuscular fat content compared with mares and geldings and higher (*p* < 0.01) cholesterol content compared with mares. The meat of stallions showed the highest pH; however, a significant (*p* < 0.01) difference was obtained only in comparison with mares. Meat lightness (L*) and yellowness (b*) of stallions were lower (*p* < 0.05) compared with geldings. Meat toughness of stallions was also lower (*p* < 0.01) than the meat of mares and geldings. The highest (*p* < 0.05 and *p* < 0.01, respectively) proportion of total polyunsaturated fatty acids (PUFA), higher (*p* < 0.05) and more favorable PUFA/SFA ratio, and also the highest (*p* < 0.05) but least favorable n-6/n-3 PUFA ratio, were detected in the muscles of stallions compared with mares and geldings. The differences in horse ages did not exhibit any effect on the properties of *M. pectoralis profundus*. Despite the age differences at the decline of horse life, the properties of the horse meat exhibited good quality and showed that horse meat is potentially highly beneficial to human health.

## 1. Introduction

Horses have long-played an important role in human society, being used for transport, draught power, and for sport and recreation, as well as for meat and milk production [1]. However, the use of horses for farm-work is decreasing, even in countries where it is still common; therefore, horse meat production may be a one of the ways to support horse populations. Eating of horse meat has always occurred, which means that a significant proportion of horses exists because of their role in the food chain [2]. In different countries, attitudes towards horse meat and its consumption habits are determined by various factors [3,4,5]. Some Western European countries are consuming and importing horse meat [3,6,7,8,9], despite the traditions and differences in consumer attitudes towards horse meat. Although the European horse meat chain is not standardized, the demand for alternative meat compared to conventional meat is increasing and thus, horse meat could play a role in this context as a red meat alternative to beef and is claimed as a dietetic meat [10]. With the aim to promote horse meat consumption by humans and encourage horse owners to send their obsolescent horses to slaughterhouses, several projects have been undertaken in some Northern countries [2]. Recently, results have been published regarding horse carcass characteristics and meat quality [7,11,12,13,14,15,16], as well as nutritional value and fatty acid composition [17,18,19,20]. However, the foal mainly represents the most studied horse slaughter category [12,13,14,18,21,22,23]. Some authors have reported that, nowadays, horse meat is generally supplied by young animals [19], while others indicated that many slaughtered horses imported to horse-meat-consuming countries were working horses from different countries [6,24]. Even in a country such as Spain, with traditions of horse meat consumption, most registered horses are raised for leisure activities and only 6.1% are used for meat production [25]. Despite having numerous horses, Lithuanians have no tradition of horse meat consumption, no domestic market for horse meat as a human food, and no specialized horse meat production systems. In Lithuania the slaughter of horses for human consumption is occurring due to the demand for horse meat by other countries and export possibilities. Different horses (females, entire males, and castrated males) come for slaughter from farms, often at the end of their use (injuries of the musculoskeletal, respiratory diseases and metabolic disturbance systems, handling, and other problems); therefore, their ages, like other characteristics, can vary quite widely. Thus, the aim of this study was to assess the influence of gender and age, and carcass weight on horse meat properties of adult horses slaughtered in Lithuania.

## 2. Materials and Methods

### 2.1. Animals and Sampling

Immediately after slaughter at an accredited abattoir, carcasses were chilled at 2–4 °C for 24 h. The samples were excised from the left of each carcass before their shipment.

With the aim to avoid the damage of the carcasses for export, the samples of *m. pectoralis profundus* were excised. Twenty-six adult horses (10 mares, 8 stallions and 8 geldings) from 3 to 21 years of age were used in the experiment. Fresh samples were used for the detection of meat proximate composition and quality properties. The samples for fatty acid composition, lipid oxidation, cholesterol content, and texture analysis were packed and immediately frozen. After being packed in plastic bags, the samples designed for the evaluation of aging effects on meat cooking loss and texture were stored for 5 days at +4 °C and frozen afterwards. All frozen samples were stored for two weeks at −65 ± 2.5 °C until analysis.

### 2.2. Meat Quality Assessments

#### 2.2.1. Proximate Composition

The dry matter content was determined [26] by drying samples in an oven at 105 °C until a constant weight was obtained (method No 950.46B; AOAC, 1990). The crude protein content was determined using the Kjeldahl method with a Kjeltec system 1002 apparatus (Foss-Tecator AB, Höganäs, Sweden), and a conversion factor of 6.25 was used to convert total nitrogen to crude protein (method No 981.10; AOAC, 1990). Crude fat was determined using the Soxhlet extraction method (method No 960.39; AOAC, 1990). Ash was determined by incineration in a muffle furnace at 550 °C for 24 h (method No 920.153; AOAC, 1990). The content of protein, fat, and ash were expressed as weight percentage of dry matter from muscle tissues.

The hydroxyproline content was determined using the NMKL-AOAC method [27].

The cholesterol content in meat was determined according to the extraction method described by Polak et al. [28], and followed by HPLC separation and analysis using a Shimadzu 10 A HPLC system (Shimadzu Corp., Kyoto, Japan). Data collection and evaluation were performed using an LC Solution (Shimadzu Corp., Kyoto, Japan) operating system. The analytical column was a LiChrospher 100 RP-18e, 150 × 4.6 mm, 5 μm (Alltech Associates Inc., Columbia, MD, USA) with a guard column (LiChrospher 100 RP-18, 7.5 × 4.6 mm). The cholesterol content was expressed as mg/100 g fresh meat.

The malondialdehyde (MDA) content in meat was measured according to the procedure of Mendes et al. [29]. MDA from well-ground meat samples was extracted with a mixture of 7.5% trichloracetic acid (TCA) solution (7.5% (p/v) TCA, 0.1% (p/v) EDTA, 0.1% (p/v) propylgallate). The extracts were deritivatized with 40 mM 2-thiobarbituric acid (TBA) solution. The MDA content was analyzed chromatographically on a Shimadzu 10 AVP HPLC system (Shimadzu Corp., Kyoto, Japan). The HPLC system consisted of a solvent delivery module (LC-10ATVP), auto injector (SIL-10ADVP), spectrofluorimetric detector (RF-10AXL), column oven (CTO-10ACVP), low pressure gradient flow control valve (FCL-10ALVP), solvent degasser (DGU-14A), and system controller (SCL-10AVP). Data collection and evaluation were performed using an LC Solution (Shimadzu Corp., Kyoto, Japan) operating system. The analytical column was a LiChrospher 100 RP-18, 250 × 4.6 mm, 5 μm (Alltech Associates Inc., USA) with a guard column (LiChrospher 100 RP-18, 7.5 × 4.6 mm). The mobile phase consisted of 50 mM KH2PO4 buffer solution, methanol, and acetonitrile in a proportion of 72:17:11 (*v*/*v*) and pumped isocratically at a flow rate of 1.0 mL/min. Spectrofluorimetric detector wave lengths were set at 525 nm (excitation) and 560 nm (emission). The results were expressed as μmol of MDA per 1 kg of muscle.

#### 2.2.2. pH Measurements

pH was measured using a digital portable pH-meter PT-380 (Boeco, Hamburg, Germany) equipped with a glass electrode (Witeg Laboratory Technik GMBH, Wertheim, Germany) after calibration using pH 4.0 and 7.0 buffer solutions. pH1 was determined 30–45 minutes post-mortem, directly on the carcass before cooling. Ultimate pH (pHu) was determined 24 h after slaughter. In LD muscle, pH was determined between the 13th and 14th last ribs at a 5-cm depth; in SM muscle, pH was determined 5 cm above the bone and at a 3-cm depth.

#### 2.2.3. Color

The color parameters were measured using a chromameter CR-410 Konica Minolta (Osaka, Japan) equipped with a 50-mm aperture using a C illuminant and 2° standard observer calibrated to a standard white calibration plate (Y = 85.3, x = 0.3173, y = 0.3251) in the CIE L* a* b* and L* C h color spaces (lightness, L*; redness, a*; yellowness, b*; chroma, C and hue, h) on a freshly cut surface after 30 min of blooming at room temperature (18 °C).

#### 2.2.4. Water Holding Capacity and Cooking Loss

Water holding capacity was measured in two ways: drip loss and cooking loss. The drip loss was assessed according to the EZ-DripLoss method. The cuts (about 20 g) were made with a fixed blade knife that was 25 mm in diameter, into muscle slices with a thickness of 25 mm and each sample was placed in a tarred special EZ-DripLoss container. The containers were closed, weighed, and left in storage at 4 °C for 24 h. The meat samples were removed from the containers after 24 h and each container with exudate meat juice was weighed again on the scale used for the tarring procedure [30]. EZ-DripLoss (%) is defined as the difference in sample weight before and after exudation. 

To determine the cooking loss, the frozen samples (120 g) for TPA and WBSF analysis were thawed at 4 °C for 24 h, weighed, and cooked in thin walled plastic bags at 80 °C for 1 h by immersion in a water bath with automatic temperature control [31], and then cooled at room temperature (18 ± 2 °C) and weighed again. The cooking loss (%) is defined as the difference in weight of the sample (after wiping dry) before and after cooking and cooling, divided by the sample weight at the beginning and multiplied by 100.

#### 2.2.5. Instrumental Evaluation of Texture

The texture of *m. pectoralis profundus* was instrumentally measured using the Warner-Bratzler shear test (WBSF) and by texture profile analysis (TPA) using a TA 1 Texture Analyser (Measurement and Calibration Technologies Ametek Comp., Lloyd instruments, Largo, FL. USA) after cooking and cooling at room temperature (20 °C). The samples for the WB test were obtained by cutting cylindrically shaped pieces of 2 × 2 cm of cross section, parallel to the muscle fiber direction. They were completely cut using a WBSF shear blade with a triangular slot cutting edge and two parameters were measured—work of shear and toughness, according to the following testing procedures: pre-test speed: 3 mm/s, test speed: 1 mm/min, post-test speed: 3 mm/s, trigger force was 10 N. The samples for TPA were prepared and analyzed by cutting cylindrically shaped pieces of 2 × 2 cm, parallel to the muscle fiber direction and then compressing to 75%. In this test, a 10 N load cell and cylindrical 20 mm-diameter probe were used. The sample was placed under the probe that moved downwards at a constant speed of 3.0 mm/s (pre-test), 1.0 mm/min (test) and 1.0 mm/s (post–test). All WBSF (work of shear and toughness) and TPA parameters (hardness, cohesiveness, springiness, and chewiness) were measured and calculated using Lloyd Instruments Ltd Nexygen/Ondio software, together with Production Test program V3.0.1. Texture parameters were tested in Newtons (N).

### 2.3. Fatty Acid Profiles

The extraction of lipids for fatty acid analysis was performed using a mixture of two volumes of chloroform (Chromasolv Plus for HPLC containing 0.5–1.0% ethanol as stabilizer) and one volume of methanol as described by Folch et al. [32]. Methylation of the samples was performed using sodium methoxide: 5 mL of 25 wt % solution in methanol was added to the sample and stirred. After 1 h, 7 mL HCL, 6 mL hexane and 2 mL H_2_O were added. The top layer was transferred into a new test-tube and evaporated. Fatty acid methyl esters were prepared according to the procedure described by Chistopherson and Glass [33]. FAMEs were analyzed using a gas liquid chromatograph (GC–2010 SHIMADZU, Kyoto, Japan) fitted with a flame ionization detector. The separation of methyl esters of fatty acids was performed on the capillary column Rt 2560 (100 m × 0.25 mm × 0.2 μm; Restek, Bellefonte, PA, USA) by temperature programming from 140 °C to 240 °C. The temperatures of the injector and detector were held, respectively, at 240 °C and 260 °C. The rate of flow of the carrier gas (nitrogen) through the column was 0.79 mL/min. The peaks were identified by comparison with the retention times of the standard fatty acid methyl esters 37 Component FAME Mix and trans FAME MIX k 110 (Supelco, Bellefonte, PA, USA). The relative proportion of each fatty acid was expressed as the relative percentage of the sum of the total fatty acids using “C solution” software for Shimadzu gas chromatograph workstations.

### 2.4. Lipid Quality Indices

Lipid quality indices, i.e., atherogenic index (AI) and thrombogenic index (TI), were calculated according to Ulbricht and Southgate [34]. The hypocholesterolemic/hypercholesterolemic (h/H) ratio was calculated according to Fernandez et al. [35]. The peroxidizability index (PI) was determined according to Du et al. [36].

### 2.5. Statistical Analysis

The data were subjected to the analysis of variance in general linear model (GLM) procedure in IBM SPSS Statistics 22 with LSD tests to determine the significance of differences of the means between groups. The GLM model included a fixed factor of gender. The age and carcass weight of the horses were included as covariates. The differences were regarded as significant when *p* < 0.05.

## 3. Results and Discussion

### 3.1. Characteristics of Slaughtered Horses

No significant differences between the groups were observed regarding horses and their carcass weights (Table 1); however, the age of horses differed considerably, because horse owners refuse of their horses for different reasons, such as age, handling problems, injures, or economical–farming changes. 

As reported by Saastamoinen [2], the slaughter of horses can be considered as an ethical way to put down horses from the point of view of animal welfare, environmental impacts, and human food production. Despite the fact that the foal represents the most studied horse slaughter category [23], a high percentage of horse meat mostly comes from culled animals [6,24]. In the present study, stallions and geldings were the youngest and oldest (*p* < 0.001), respectively. A Swedish study [37] on horse longevity and causes of culling indicated a longer median length of life for cold blood geldings than for mares and this tendency could be also observed in the present study for the slaughtered horses.

### 3.2. Meat Quality

Although the results of other authors were obtained on foals and showed the effect of muscle anatomical location on many different meat quality parameters [38], in general all muscles presented high levels of protein (>21.2%). In the present study, the *m. pectoralis profundus* of older horses than those in the study mentioned above exhibited slightly higher protein contents (Table 2). Other authors [39] who studied the influence of age on meat quality indicated that horse meat from younger animals, from 4 to 7 years of age, had less protein and fat compared with the older group, from 8 to 12 years. Moreover, older donkeys that were 18 months of age also had higher intramuscular fat and meat protein contents compared with younger, 12 months-old donkey foals [40]. Although the differences in horse age did not affect the proximate composition of *m. pectoralis profundus,* the horse gender appeared to affect dry matter, fat, and cholesterol contents in horse meat. The stallions had lower (*p* < 0.01 and *p* < 0.05, respectively) content of dry matter and also considerably lower (*p* < 0.001 and *p* < 0.01) intramuscular fat content compared with mares and geldings.

The stallions also had higher (*p* < 0.01) cholesterol content compared with mares. However, all the differences between the mares and geldings were insignificant. Lorenzo and Pateiro [18] reported that cholesterol content did not show any significant differences among different muscles of fifteen-month-old foals with a mean value range between 0.57 and 0.62 mg/100 g; however, Seong et al. [41] showed the effect of different retail cuts on the cholesterol content with a value range between 55.76 and 79.5 mg/100 g, and these values were higher than those in the present study for the *m. pectoralis profundus* of adult horses. Higher cholesterol levels (62.4–63.9 mg/100g) were also found in the meat of young donkeys [42]. Furthermore, *m. longissimus dorsi* of 9–11-year-old geldings slaughtered in Brazil [43] had lower (40 mg/100 g) cholesterol and IMF contents than the geldings in the present study.

Analysis of the properties of *m. pectoralis profundus* indicated that horse gender affected, or tended to affect, meat pH and color parameters (Table 3).

The meat of stallions showed the highest pH; however, a significant (*p* < 0.01) difference was obtained only in comparison with mares. Meat lightness (L*) and yellowness (b*) of stallions were lower (*p* < 0.05) compared with geldings. The hue angle (h) of stallion meat was lower (*p* < 0.05 and *p* < 0.01, respectively) than that of mares and geldings. Sarries and Beriain [44] found that sixteen-month-old females had lighter muscles than males and explained that the darker meat of males could be due to their greater physical activity. Other authors [45] did not find any significant differences between female and male foals at fifteen months of age, except for the cooking loss, where the males presented higher values. Although insignificant, higher cooking loss values were also demonstrated by the stallions in the present study.

The *m. pectoralis profundus* of the stallions before aging was characterized by the lowest (*p* < 0.01) toughness with the Warner–Bratzler test, however, a significant difference was found only between stallions and mares (Table 4).

Most authors [13,14,15,16,22,45] declared no effect by horse foal sex and age on WBSF, whereas shear force was lower in donkeys slaughtered at 8 months of age compared to older animals [42]. Litwinczuk et al. [46] found an effect of the muscle of 10-year-old horses on horse meat parameters, including Warner–Bratzler shear force (WBSF). However, *m. pectoralis profundus* has not been evaluated by any authors. WBSF decreased during aging and this is in agreement with authors [47,48] who showed lower WBSF values in different muscles after aging.

Neither the gender nor the age of horses showed any effect on the parameters of the texture profile analysis (TPA) and this is in agreement with the findings for foal meat; however, the weight of the carcass appeared to affect (*p* < 0.05) the springiness and cohesiveness in fresh meat and in meat after aging, respectively (Table 5). There is an opinion that TPA assessment could predict sensory hardness of raw meat better than WBSF examination, but for cooked meat the WBSF method is better [49]. In the present study, both WBSF and TPA tests were used on cooked meat and it seems that the WBSF test was better at showing the effect of aging on horse meat tenderness.

### 3.3. Fatty Acid Composition

No significant differences between the genders were observed for the total saturated fatty acids (SFA), but for saturated fatty acids, stallions had higher (*p* < 0.05 and *p* < 0.01, respectively) proportions of individual C17:0 and C18:0 fatty acids compared with mares and geldings (Table 6) and similarly lower (*p* < 0.05) proportions of C14:0 than geldings. Geldings had the highest (*p* < 0.05) proportion of C15:0.

Many authors who investigated the fatty acid composition of young foal meat [14,16,20,22,45,48] reported higher proportions of SFA than those found in old horse meat in the present study. Moreover, no significant differences have been found, either between males and females or between age groups. Higher proportions than in the present study of SFA were also reported for 30–36-month-old stallions and geldings of the Jeju horse breed [50].

The highest proportion of total monounsaturated fatty acids (MUFA) was found in the muscles of geldings (Table 7) and this is in agreement with the data presented by He et al. [51]. Geldings showed higher (*p* < 0.05) proportion of MUFA, including C17:1n-9, C18:1n-9, C18:1trans-9, and also a higher (*p* < 0.01) proportion of C16:1n-7 compared with stallions.

Mares also had higher (*p* < 0.01) proportions of total MUFA, including C17:1n-9 and C22:1n-9, and a higher (*p* < 0.05) proportion of C16:1n-7, but lower (*p* < 0.01) proportions of C16:1trans-9 and C18:1trans-9, than stallions. Higher proportions of MUFA in the meat from mares compared with that of stallions is consistent with the findings of Tateo et al. [14]; however, other authors [17,45] did not indicate any significant differences between foal sex. In agreement with our study, some other authors [22] also did not find any effects of animal age on MUFA, whereas others [16] reported that MUFA proportion increased and was associated with foal age increase.

The highest (*p* < 0.05 and *p* < 0.01, respectively) proportion of total polyunsaturated fatty acids (PUFA), including individual C18:2n-6, C20:3n-3, C20:4n-6, and C22:5n-3 acids, was detected in the muscles of stallions compared with mares and geldings (Table 8). Higher proportions of PUFA were also found in the meat of male foals compared with female foals [14]; however, other authors [17,45] did not observed any sex effect on foal meat PUFA. Stallions also had higher (*p* < 0.05 and *p* < 0.01, respectively) proportions of C20:2n-6, C22:4n-6 and C20:3n-6, C22:6n-3 than mares; however, higher (*p* < 0.05 and *p* < 0.01, respectively) proportions of ALA (C18:3n-3) and EPA (C20:5n-3) were found in the muscles of mares compared with stallions. Although authors who compared horse meat with beef and pork [50,51] declared that horses had lower SFA and MUF, but higher PUFA compared with cattle and pigs, in their studies, the muscles of fattened horses had lower PUFA proportions (13.5–16.76%) than those found in the present study.

The effect of gender was observed for trans fatty acid isomers (Table 9). The stallions had higher (*p* < 0.01 and *p* < 0.05, respectively) contents of total trans fatty acid isomers (TFA) compared with mares and geldings. 

The PUFA/SFA ratio in horse meat was above the minimum (0.4) recommended for the consumer diet [52]. A higher (*p* < 0.05) and more favorable PUFA/SFA ratio was found in the muscles of stallions than in the muscles of mares and geldings. Lorenzo et al. [45] determined a higher PUFA/SFA ratio (1.0); however, they did not find any differences between foal males and females at fifteen months of age. 

The PUFA/SFA ratios estimated in the present study for the meat from mares and geldings were similar to those reported by other authors [7,22,41] and considerably more favorable than those found for Jeju horses [50].

As the recommendations of Bellagio’s report on healthy agriculture, healthy nutrition and healthy people indicate that a ratio (4:1) of n-6 PUFA to n-3 PUFA in the diet should be the goal [53], it can be observed that n-6/n-3 PUFA ratios in horse meat of all genders were excellent. Nevertheless, the highest (*p* < 0.05) and least favorable n-6/n-3 PUFA ratio was observed for stallions compared with mares and geldings. Although age did not appear to affect the fatty acid composition and fatty acid ratios, younger fifteen-month-old males in the study of Lorenzo et al. [45] demonstrated a lower (1.8) n-6/n-3 PUFA ratio than the adult stallions in the present study. However, n-6/n-3 PUFA ratios of horse meat from mares and geldings in the present study were lower and more favorable than those ratios of the horse meat from young animals in different studies [22,41,45].

The differences in fatty acid composition between the horse genders appeared to affect (*p* < 0.05) thrombogenic (TI) and peroxidizability (PI) indexes in the IMF. The stallions showed higher (*p* < 0.01) and less favorable TI and PI indexes than the mares. Sarries et al. [12] also found a higher TI index for younger males. Different authors [12,17,20] found higher AI and TI indexes, but a lower h/H ratio for the meat of foals compared with those in the present study. Although horse meat showed a similar AI index and h/H ratio compared with the pork from Lithuanian pig breeds [54], it must be noted that horse meat demonstrated a considerably lower TI index.

## 4. Conclusions

The analysis model applied in this study did not exhibit any effect of adult horse age on the properties of *m. pectoralis profundus*. Gender appeared to affect horse meat properties. Stallions demonstrated the largest differences regarding the content of dry matter, intramuscular fat, and cholesterol content, meat pH and color measures, toughness, fatty acid composition in intramuscular fat, fatty acid ratios and lipid quality indices compared with mares and geldings. Carcass weight appeared to affect the fat content, horsemeat lightness (L*), hue (h), proportions of total saturated (SFA) and polyunsaturated (PUFA) fatty acids, PUFA/SFA ratio, atherogenic index (AI) and hypocholesterolemic/hypercholesterolemic (h/H) ratio. Despite horse age differences at the decline of life, the properties of horse meat exhibited good quality and showed that horse meat is potentially highly beneficial to human health. This study is one step towards the development and utilization of horses. The obtained information can be used to increase the diversity in meat production and consumption, and it provides new insights for research on the usage of obsolescent horses. Further additional investigations of age and other factors on meat quality from obsolescent horses are needed.

## Figures and Tables

**Table 1:** Characteristics of slaughtered horses.

Variables	Gender		
	Mares (*n* = 10)	Stallions (*n* = 8)	Geldings (*n* = 8)
Horse weight, kg	663.5 ± 37.39	671.9 ± 33.39	586.4 ± 31.52
Carcass weight, kg	384.7 ± 25.13	387.9 ± 19.79	346.4 ± 19.81
Age, years	8.5 ^a,e^ ± 1.26	3.4 ^b,e^ ± 1.41	17.7 ^f^ ± 1.50

The differences between the means of genders in the rows with different superscripts differ at a, b: *p* *˂* 0.05; e, f: *p* < 0.001.

**Table 2:** Effects of horse gender, age, and carcass weight on proximate composition of *m*. *pectoralis profundus* muscle.

Variables	Gender			*p*-Value		
	Mares *n* = 10	Stallions *n* = 8	Geldings *n* = 8	Gender	Weight	Age
Dry matter, %	28.22 ^c^ ± 0.50	25.46 ^a,d^ ± 0.75	28.76 ^b^ ± 0.92	0.014	0.456	0.164
Protein, %	21.39 ± 0.32	21.98 ± 0.49	21.57 ± 0.60	0.539	0.139	0.900
Fat,%	4.92 ^e^ ± 0.51	1.57 ^f,c^ ± 0.77	6.08 ^d^ ± 0.95	0.004	0.007	0.141
Ash, %	0.85 ± 0.04	0.95 ± 0.06	0.82 ± 0.07	0.346	0.187	0.988
Hydroxyproline, mg/100g	159.82 ± 11.30	149.18 ± 17.04	172.01 ± 20.98	0.786	0.837	0.772
Cholesterol, mg/100 g	40.05 ^c^ ± 2.55	52.75 ^d^ ± 3.85	45.06 ± 4.74	0.018	0.315	0.538
MDA	0.80 ± 0.19	0.63 ± 0.35	0.54 ± 0.31	0.703	0.051	0.291
MDA after aging	3.15 ± 0.89	0.92 ± 1.69	3.20 ± 1.47	0.443	0.516	0.601

The differences between the means of genders in the rows with different superscripts differ at a, b: *p* *˂* 0.05; c, d: *p* ˂ 0.01; e, f: *p* < 0.001; MDA = malondialdehyde.

**Table 3:** Effects of horse gender, age and carcass weight on meat quality parameters.

Variables	Gender			*p*-Value		
	Mares *n* = 10	Stallions *n* = 8	Geldings *n* = 8	Gender	Weight	Age
pH	5.73 ^c^ ± 0.10	6.25 ^d^ ± 0.15	5.74 ± 0.18	0.018	0.699	0.535
Color: L*	33.50 ± 0.52	31.91 ^a^ ± 0.78	35.40 ^b^ ± 0.96	0.094	0.001	0.516
a*	19.81 ± 1.05	17.35 ± 1.58	22.11 ± 1.95	0.295	0.332	0.204
b*	6.40 ± 0.61	4.49 ^a^ ± 0.92	8.88 ^b^ ± 1.14	0.072	0.051	0.216
C	20.86 ± 1.16	17.85 ± 1.74	23.80 ± 2.15	0.217	0.241	0.197
h	17.40 ^a^ ± 0.83	14.41 ^b,c^ ± 1.26	21.97 ^d^ ± 1.55	0.021	0.005	0.585
Drip loss,%	1.22 ± 0.22	0.77 ± 0.33	0.43 ± 0.40	0.126	0.259	0.932
Cooking loss,%	40.27 ± 1.38	41.38 ± 2.09	37.48 ± 2.57	0.627	0.217	0.526
Cooking loss after aging,%	44.76 ± 0.70	46.13 ± 1.33	43.66 ± 1.16	0.510	0.638	0.322

The differences between the means of genders in the rows with different superscripts differ at a, b: *p* *˂* 0.05; c, d: *p* ˂ 0.01.

**Table 4:** Effects of horse gender, age, and carcass weight on Warner–Bratzler shear force parameters in cooked *pectoralis profundus* muscle, before and after aging.

Variables	Gender			*p*-Value		
	Mares *n* = 10	Stallions *n* = 8	Geldings *n* = 8	Gender	Weight	Age
Shear of force, N	4.96 ± 0.68	3.38 ± 1.03	6.01 ± 1.26	0.357	0.555	0.849
Toughness, N	976.69 ^c^ ± 93.12	433.74 ^d^ ± 140.40	963.37 ± 172.90	0.009	0.883	0.632
Shear of force after aging, N	4.34 ± 0.56	5.57 ± 1.07	6.24 ± 0.93	0.151	0.912	0.754
Toughness after aging, N	281.35 ± 25.53	330.19 ± 48.69	363.66 ± 42.32	0.196	0.265	0.901

The differences between the means of genders in the rows with different superscripts differ at c, d: *p* ˂ 0.01.

**Table 5:** Effects of horse gender, age and carcass weight on parameters of texture profile analysis in cooked *m*. *pectoralis profundus* muscle before and after aging.

Variables	Gender			*p*-Value		
	Mares *n* = 10	Stallions *n* = 8	Geldings *n* = 8	Gender	Weight	Age
Before aging						
Cohesiveness	2.67 ± 0.11	2.45 ± 0.17	2.17 ± 0.21	0.074	0.057	0.175
Gumminess	19.94 ± 2.62	18.19 ± 3.94	28.93 ± 4.86	0.317	0.141	0.158
Hardness, N	52.20 ± 5.83	43.93 ± 8.79	63.13 ± 10.83	0.542	0.364	0.354
Springiness, N	0.85 ± 0.01	0.85 ± 0.01	0.86 ± 0.01	0.899	0.011	0.457
Chewiness, N	17.14 ± 2.30	15.43 ± 3.47	24.80 ± 4.27	0.335	0.115	0.153
After aging						
Cohesiveness	2.70 ± 0.11	2.91 ± 0.20	2.58 ± 0.18	0.563	0.016	0.341
Gumminess, N	27.43 ± 2.77	23.52 ± 5.28	27.99 ± 4.59	0.769	0.082	0.899
Hardness, N	71.75 ± 5.90	69.04 ± 11.24	70.26 ± 9.77	0.962	0.390	0.794
Springiness, N	0.85 ± 0.01	0.85 ± 0.01	0.86 ± 0.01	0.276	0.363	0.404
Chewiness, N	23.19 ± 2.33	20.04 ± 4.45	24.09 ± 3.87	0.782	0.072	0.943

**Table 6:** Effects of gender, age, and carcass weight on saturated fatty acid (% of total FA) composition of horse meat lipids.

Fatty Acids	Gender			*p*-Value		
	Mares *n* = 10	Stallions *n* = 8	Geldings *n* = 8	Gender	Weight	Age
C10:0	0.01 ± 0.01	0.02 ± 0.01	0.03 ± 0.02	0.440	0.308	0.191
C12:0	0.10 ± 0.02	0.12 ± 0.03	0.13 ± 0.04	0.667	0.191	0.666
C14:0	2.96 ± 0.18	2.43 ^a^ ± 0.27	3.62 ^b^ ± 0.34	0.102	0.058	0.708
C15:0	0.18 ^a^ ± 0.01	0.15 ^a^ ± 0.02	0.24 ^b^ ± 0.02	0.061	0.035	0.099
C16:0	24.69 ± 0.60	23.28 ± 0.90	26.07 ± 1.11	0.280	0.028	0.895
C17:0	0.21 ^b^ ± 0.02	0.29 ^a^ ± 0.03	0.16 ^b^ ± 0.04	0.087	0.981	0.908
C18:0	3.65 ^d^ ± 0.50	6.35 ^c^ ± 0.75	2.09 ^d^ ± 0.93	0.012	0.149	0.552
C20:0	0.06 ± 0.00	0.07 ± 0.01	0.05 ± 0.01	0.331	0.113	0.892
C22:0	0.02 ± 0.00	0.01 ± 0.00	0.01 ± 0.01	0.070	0.892	0.290
SFA	31.88 ± 0.50	32.72 ± 0.75	32.41 ± 0.93	0.533	0.041	0.818

The differences between the means of genders in the rows with different superscripts differ at a, b: *p* *˂* 0.05; c, d: *p* ˂ 0.01; SFA = sum of all identified saturated fatty acids.

**Table 7:** Effects of gender, age, and carcass weight on monounsaturated fatty acid (% of total FA) composition of horse meat lipids.

Fatty Acids	Gender			*p*-Value		
	Mares *n* = 10	Stallions *n* = 8	Geldings *n* = 8	Gender	Weight	Age
C14:1n-7	0.27 ± 0.04	0.20 ± 0.06	0.41 ± 0.08	0.222	0.189	0.401
C15:1	0.05 ± 0.01	0.03 ± 0.02	0.01 ± 0.02	0.190	0.595	0.380
C16:1n9trans	0.04 ^c^ ± 0.01	0.08 ^d^ ± 0.01	0.04 ± 0.02	0.021	0.270	0.650
C16:1n-9	0.54 ± 0.07	0.44 ± 0.11	0.64 ± 0.13	0.618	0.084	0.580
C16:1n-7	7.50 ^a^ ± 0.68	4.62 ^b,c^ ± 1.03	10.64 ^d^ ± 1.26	0.021	0.117	0.335
C17:1n-9	0.60 ^c^ ± 0.04	0.34 ^d,a^ ± 0.07	0.67 ^b^ ± 0.08	0.011	0.651	0.534
C18:1n-9trans	0.08 ^c^ ± 0.01	0.12 ^d,a^ ± 0.01	0.07 ^b^ ± 0.02	0.025	0.214	0.915
C18:1n-9	31.28 ^a^ ± 1.59	23.86 ^b^ ± 2.40	31.66 ± 2.95	0.041	0.155	0.498
C18:1n-7	2.13 ± 0.07	1.93 ^a^ ± 0.11	2.42 ^b^ ± 0.14	0.092	0.034	0.154
C20:1n-9	0.40 ± 0.02	0.35 ± 0.03	0.39 ± 0.03	0.188	0.874	0.549
C22:1n-9	0.03 ^c^ ± 0.00	0.01 ^d^ ± 0.01	0.02 ± 0.01	0.078	0.411	0.102
MUFA	42.90 ^c^ ± 1.99	31.96 ^d,a^ ± 2.99	46.98 ^b^ ± 3.69	0.013	0.119	0.357

The differences between the means of genders in the rows with different superscripts differ at a, b: *p* *˂* 0.05; c, d: *p* ˂ 0.01; MUFA = sum of all identified monounsaturated fatty acids.

**Table 8:** Effects of gender, age, and carcass weight on polyunsaturated fatty acid (% of total FA) composition of horse meat lipids.

Fatty Acids	Gender			*p*-Value		
	Mares *n* = 10	Stallions *n* = 8	Geldings *n* = 8	Gender	Weight	Age
c9,t12 C18:2	0.02 ± 0.01	0.04 ± 0.01	0.01 ± 0.01	0.183	0.343	0.450
C18:2n-6	8.97 ^c^ ± 1.40	16.26 ^d,a^ ± 2.11	7.08 ^b^ ± 2.60	0.021	0.059	0.544
C18:3n-6	0.09 ± 0.01	0.10 ± 0.02	0.06 ± 0.02	0.438	0.166	0.291
C18:3n-3	13.31 ^a^ ± 0.97	9.09 ^b^ ± 1.47	12.91 ± 1.81	0.056	0.476	0.970
C20:2n-6	0.16 ^a^ ± 0.02	0.23 ^b^ ± 0.03	0.15 ± 0.04	0.114	0.173	0.831
C20:3n-6	0.12 ^c^ ± 0.07	0.44 d ± 0.11	0.20 ^c^ ± 0.14	0.020	0.112	0.534
C20:3n-3	0.33 ^c^ ± 0.04	0.54 ^d,a^ ± 0.06	0.26 ^b^ ± 0.07	0.023	0.146	0.436
C20:4n-6	0.44 ^c^ ± 0.28	1.78 ^d,a^ ± 0.43	0.37 ^b^ ± 0.53	0.016	0.093	0.503
C20:5n-3	0.75 ^c^ ± 0.04	0.29 ^d,a^ ± 0.06	0.06 ^b^ ± 0.08	0.027	0.164	0.478
C22:2n-6	0.05 ± 0.01	0.07 ± 0.01	0.04 ± 0.01	0.488	0.144	0.354
C22:4n-6	0.02 ^a^ ± 0.01	0.07 ^b^ ± 0.02	0.02 ± 0.02	0.064	0.466	0.975
C22:5n-3	0.26 ^c^ ± 0.11	0.84 ^d,a^ ± 0.17	0.07 ^b^ ± 0.20	0.018	0.099	0.460
C22:6n-3	0.04 ^c^ ± 0.02	0.16 ^d,a^ ± 0.03	0.00 ^b^ ± 0.04	0.013	0.076	0.466
PUFA	23.98 ^b^ ± 1.46	30.25 ^a^ ± 2.21	20.53 ^b^ ± 2.72	0.051	0.044	0.374

The differences between the means of genders in the rows with different superscripts differ at a, b: *p* *˂* 0.05; c, d: *p* ˂ 0.01; PUFA = sum of all identified polyunsaturated fatty acids.

**Table 9:** Total trans fatty acids and fatty acid ratios and lipid quality indexes in intramuscular fat of horse meat.

Variables	Gender			*p*-Value		
	Mares *n* = 10	Stallions *n* = 8	Geldings *n* = 8	Gender	Weight	Age
TFA	0.14 ^c^ ± 0.02	0.25 ^d,a^ ± 0.03	0.11 ^b^ ± 0.03	0.006	0.133	0.617
PUFA/SFA	0.76 ^b^ ± 0.05	0.94 ^a^ ± 0.08	0.62 ^b^ ± 0.10	0.094	0.022	0.371
n-6/n-3 PUFA	0.73 ^c^ ± 0.30	2.17 ^d^ ± 0.45	0.53 ± 0.56	0.034	0.224	0.794
AI	0.55 ± 0.02	0.53 ± 0.03	0.61 ± 0.03	0.338	0.019	0.952
TI	0.45 ^c^ ± 0.02	0.56 ^d^ ± 0.03	0.47 ± 0.04	0.028	0.811	0.983
h/H	2.09 ± 0.07	2.22 ± 0.10	1.81 ± 0.13	0.149	0.019	0.636
PI	42.54 ^b^ ± 2.76	54.42 ^a^ ± 4.15	35.19 ^b^ ± 5.12	0.045	0.055	0.345

The differences between the means of genders in the rows with different superscripts differ at a, b: *p* *˂* 0.05; c, d: *p* ˂ 0.01; TFA = sum of all identified trans fatty acids; PUFA/SFA = ratio of PUFA to SFA, n-6/n-3 = ratio of n-6 PUFA to n-3 PUFA, AI = atherogenic index, TI = thrombogenic index, h/H = hypocholesterolemic/hypercholesterolemic ratio, PI = peroxidizability index.

## Data Availability

Data are contained within this article.
